# Altered Coupling Between Cerebral Blood Flow and Voxel-Mirrored Homotopic Connectivity Affects Stroke-Induced Speech Comprehension Deficits

**DOI:** 10.3389/fnagi.2022.922154

**Published:** 2022-06-23

**Authors:** Jie Zhang, Desheng Shang, Jing Ye, Yi Ling, Shuchang Zhong, Shuangshuang Zhang, Wei Zhang, Li Zhang, Yamei Yu, Fangping He, Xiangming Ye, Benyan Luo

**Affiliations:** ^1^Center for Rehabilitation Medicine, Rehabilitation & Sports Medicine Research Institute of Zhejiang Province, Department of Rehabilitation Medicine, Zhejiang Provincial People’s Hospital (Affiliated People’s Hospital, Hangzhou Medical College), Hangzhou, China; ^2^Department of Neurology, Brain Medical Center, The First Affiliated Hospital of Zhejiang University School of Medicine, Hangzhou, China; ^3^Department of Radiology, The First Affiliated Hospital of Zhejiang University School of Medicine, Hangzhou, China; ^4^Department of Neurology, Sir Run Run Shaw Hospital, Zhejiang University School of Medicine, Hangzhou, China; ^5^Collaborative Innovation Center for Brain Science, Zhejiang University School of Medicine, Hangzhou, China

**Keywords:** ischemic stroke, speech comprehension, cerebral blood flow, arterial spin labeling, homotopic connectivity, neurovascular coupling, functional magnetic resonance imaging

## Abstract

The neurophysiological basis of the association between interhemispheric connectivity and speech comprehension processing remains unclear. This prospective study examined regional cerebral blood flow (CBF), homotopic functional connectivity, and neurovascular coupling, and their effects on comprehension performance in post-stroke aphasia. Multimodal imaging data (including data from functional magnetic resonance imaging and arterial spin labeling imaging) of 19 patients with post-stroke aphasia and 22 healthy volunteers were collected. CBF, voxel-mirrored homotopic connectivity (VMHC), CBF-VMHC correlation, and CBF/VMHC ratio maps were calculated. Between-group comparisons were performed to identify neurovascular changes, and correlation analyses were conducted to examine their relationship with the comprehension domain. The correlation between CBF and VMHC of the global gray matter decreased in patients with post-stroke aphasia. The total speech comprehension score was significantly associated with VMHC in the peri-Wernicke area [posterior superior temporal sulcus (pSTS): *r* = 0.748, *p* = 0.001; rostroventral area 39: *r* = 0.641, *p* = 0.008]. The decreased CBF/VMHC ratio was also mainly associated with the peri-Wernicke temporoparietal areas. Additionally, a negative relationship between the mean CBF/VMHC ratio of the cingulate gyrus subregion and sentence-level comprehension was observed (*r* = −0.658, *p* = 0.006). These findings indicate the contribution of peri-Wernicke homotopic functional connectivity to speech comprehension and reveal that abnormal neurovascular coupling of the cingulate gyrus subregion may underly comprehension deficits in patients with post-stroke aphasia.

## Introduction

Auditory comprehension deficits are among the most devastating consequences of stroke, and post-stroke problems in communication can severely impair the patients’ quality of life and make even simple daily tasks challenging. The human auditory system is structurally and functionally asymmetric; this asymmetry is an important prerequisite for phonological processing in humans (Specht, 2014). Meaningful messages can be recognized among continuously changing acoustic signals through auditory speech processing (Price, 2010). Over the past decades, studies have established that the superior temporal gyrus (STG) is activated bilaterally during prelexical processing, whereas left-lateralized responses are detected during the noise merging into speech (Specht et al., 2009). The lateralization theory of speech is consistent with clinical observations and voxel-based morphology studies that have reported speech comprehension deficits in post-stroke patients with dominant-hemispheric lesions (Leff et al., 2009).

However, compared with the dorsal stream for production, the ventral stream for comprehension is organized more bilaterally within the well-established framework of dual-stream language processing (Hickok and Poeppel, 2007). This hypothesis has been supported by anatomical evidence in a fiber tracking study (Rilling et al., 2011). Interhemispheric connectivity strength between bilateral temporal regions has been proposed to associate with word- and sentence-level speech comprehension performance in aphasic stroke patients (Warren et al., 2009). Moreover, not only stroke-induced disruptions in intrahemispheric segregation but also interhemispheric integration of distributed brain networks could predict stroke-induced impairments in neurobehavioral domains, including language (Siegel et al., 2016). These findings further indicate that interhemispheric connections linking homotopic cortical regions may contribute to the functional connectivity between bilateral temporal cortices during normal narrative speech comprehension. Nevertheless, interhemispheric correlated responses are less likely to be caused by simple parallel relaying of auditory information along auditory processing pathways in bilateral hemispheres (Warren et al., 2009). Thus, the mechanisms underlying interhemispheric connectivity for speech comprehension require further investigation using novel neuroimaging tools.

Homotopic resting-state functional connectivity (RSFC), a critical intrinsic feature of the functional brain architecture, can be used to depict effective interhemispheric communication (Salvador et al., 2008; Stark et al., 2008). It can be robustly quantified using voxel-mirrored homotopic connectivity (VMHC), which computes the RSFC between each voxel and its left-to-right mirrored counterpart (Zuo et al., 2010). VMHC reflects the synchrony of spontaneous neural activity in bilateral hemispheric architecture between symmetrical regions (Salvador et al., 2005a,b). Previous studies have evaluated the predictive value of VMHC in post-stroke patients (Yang et al., 2017; Shan et al., 2018; Chen et al., 2021), and their findings indicated that functional recovery was related to intrinsic alterations in interhemispheric coordination (Chen et al., 2021). In addition, VMHC values were correlated with the severity of global aphasia (Yang et al., 2017). Nonetheless, the aforementioned studies on VMHC in post-stroke patients had only utilized single-modal functional imaging. Furthermore, the neurophysiological mechanisms underlying the relationship between homotopic connectivity and functional performance are not fully understood, and the association between VMHC and specific domains such as comprehension remains to be investigated.

Emerging evidence shows that resting-state brain functional topology is tightly coupled with cerebral blood flow (CBF) (Liang et al., 2013), suggesting that regional CBF is a quantitative index of metabolic activity within specific networks (Jann et al., 2015). Neurovascular coupling exhibits the tight linkage between neural activity and hemodynamic levels (Phillips et al., 2016). Previous studies have applied the CBF/RSFC ratio in various neuropsychiatric disorders and revealed that the amount of blood supply per unit of connectivity strength is abnormal in cognitive-related brain regions (Zhu et al., 2017; Guo et al., 2019). Regarding cerebrovascular diseases, a recent study involving patients with ischemic cerebrovascular disorders has shown that neurovascular uncoupling between resting-state regional homogeneity and CBF might be related to cognitive deficits (Liu et al., 2021). Neurovascular disassociation has been confirmed to be the most severe during the subacute period of stroke and could affect the area beyond the site of initial infarction (He et al., 2020). Along with cerebral autoregulation, neurovascular coupling is necessary for the maintenance of cognitive function and may be a critical physiological mechanism for post-stroke recovery (Beishon and Minhas, 2021). Therefore, interpreting post-stroke language impairment by investigating the linkage between homotopic RSFC alterations and regional cerebral perfusion is promising.

Accordingly, the present study aimed to explore the relationships between homotopic connectivity, hemodynamics, and speech comprehension in post-stroke aphasia. We hypothesized that (1) the functional connectivity strength between homotopic regions might be related to the level of preserved speech comprehension beyond the influence of primary local ischemic injury and (2) speech comprehension deficits might be associated with altered neurovascular characteristics in brain areas.

## Materials and Methods

### Participants

This study is part of an ongoing cohort evaluation at the First Affiliated Hospital of Zhejiang University School of Medicine (Hangzhou, China), and there is thus a partial overlap with our previous work (Zhang et al., 2022). A total of 19 patients with post-stroke aphasia (14 men and 5 women; age: 60.1 ± 13.1 years) and 22 healthy participants (12 men and 10 women; age: 54.3 ± 13.7 years) with matched demographic features were prospectively enrolled. The inclusion criteria for this cohort were as follows: (1) a single left-hemisphere ischemic stroke; (2) a diagnosis of aphasia; (3) at the early subacute phase defined by the first Stroke Recovery and Rehabilitation Roundtable (Bernhardt et al., 2017) (a period after the acute stage that is characterized by a declined influence of peri-infarct edema and inflammation but is considered to be still relatively early after stroke and useful for identifying predictive biomarkers); (4) age of 18–80 years; (5) education level >6 years; (6) right-handedness; and (7) Chinese as the first language. The exclusion criteria were as follows: (1) a previous diagnosis of other neurological/psychiatric diseases; (2) evidence of alcohol or psychoactive substance abuse; and (3) contraindications for MRI. Detailed clinical characteristics are presented in Table 1.

**TABLE 1:** Demographic and clinical features of participants in different groups (*n* = 41).

Characteristics	Patients (*n* = 19)	Controls (*n* = 22)	Statistics
**Demographic characteristics**			
Sex, female, *n* (%)	5 (26.3)	10 (45.5)	χ^2^(1) = 1.610, *p* = 0.205
Age (mean ± SD, years)	60.1 ± 13.1	54.3 ± 13.7	*t*(39) = 1.377, *p* = 0.176
Education (mean ± SD, years)	9.9 ± 3.7	10.4 ± 4.5	*Z* = −0.377, *p* = 0.706
**Stroke characteristics**			
Post-stroke time (mean ± SD, days)	11.5 ± 5.7	–	–
Lesion volume (mean ± SD, mL)	61.0 ± 56.7	–	–
**Imaging characteristics**			
Intracranial volume (mean ± SD, cm^3^)	1559.3 ± 149.3	1473.8 ± 157.3	*t*(39) = 1.776, *p* = 0.084
Frame-wise displacement (mm)	0.096 ± 0.036	0.097 ± 0.062	*t*(39) = 0.052, *p* = 0.959
**Dominant hand**			
Right, *n* (%)	19 (100)	22 (100)	–
**Language scores**			
Aphasia quotient	38.8 ± 23.1	–	–
Total speech comprehension (%)	55.4 ± 23.8	–	–
Word-level speech comprehension (%)	54.2 ± 27.3	–	–
Sentence-level speech comprehension (%)	55.1 ± 21.7	–	–

*Two-sample independent t-test for age and intracranial volume, non-parametric test for years of education, and χ^2^ test for sex. SD, standard deviation.*

The studies involving human participants were reviewed and approved by the Institutional Ethics Committee of the First Affiliated Hospital of Zhejiang University School of Medicine. All study procedures were conducted in accordance with the principles of the Declaration of Helsinki. All participants provided written informed consent prior to study enrollment.

### Neuropsychological Assessment

The language performance of all patients was assessed using the Aphasia Battery of Chinese (Gao et al., 1992). A professional researcher (JY) performed the evaluation. For this study, we extracted auditory comprehension scores and their subdomains. The aphasia quotient (AQ) was also calculated to measure the overall severity of language impairment. All behavioral scores were transformed into a 100-point scale. Another experienced research assistant (YY) blindly examined and recorded the neuropsychological profiles.

### MRI Protocol

Multi-model MRI was performed using a General Electric 3T scanner. All participants were instructed to keep their head still and eyes closed, avoid specific thinking, stay awake, and relax. The scanning protocols included (1) T1-weighted imaging obtained using a 3D brain volume imaging sequence (TR/TE = 7.8/3.0 ms; voxel size = 1 mm × 0.5 mm × 0.5 mm; matrix = 256 × 256; FOV = 256 mm × 256 mm; flip angle = 7°; and no gap between slices); (2) resting-state blood oxygenation level dependent images acquired using a gradient-echo echo-planar imaging sequence (TR/TE = 2000/30 ms; voxel size = 4 mm × 3.4 mm × 3.4 mm; matrix = 64 × 64; FOV = 220 mm × 220 mm; flip angle = 7°; 180 volumes; and 33 interleaved transverse slices with interslice gap = 0.6 mm); (3) 3D arterial spin labeling (ASL) imaging of a spin-echo pulse sequence utilized to measure perfusion level (TR/TE = 4560/9.8 ms; post-label delay = 1525 ms; voxel size = 4 mm × 1.9 mm × 1.9 mm; matrix = 128 × 128; FOV = 240 mm × 240 mm; no gap between slices; spiral in readout of eight arms; and k-space sample points = 512).

### Imaging Processing

#### Generation of Lesion Maps

Two skilled neurologists (JZ and SZ) manually drew the stroke lesion masks on T1-weighted images using the ITK-SNAP software^1^ (Yushkevich et al., 2006). The volumes of individual lesions were calculated by outputting the volumes and numbers of non-zero voxels in lesion mask files using the *fslstats* command with the *-V* option in FMRIB Software Library (FSL) (v6.0.2) (Jenkinson et al., 2012). The lesion masks of all participants were transformed into the standard Montreal Neurological Institute (MNI) space, and the overlap map of lesion masks was subsequently obtained using the *fslmaths* command with the *-add* option in FSL. The lesion volume was regarded as a critical additional covariate to moderate the influence of association between language impairment and the extent of primary ischemic damage.

#### T1-Weighted Structural Image Preprocessing

T1-weighted structural images were preprocessed to obtain transformation matrices for subsequent co-registration. The *MR segment-normalize* module of the Clinical Toolbox^2^ was utilized for patients’ T1-weighted images. Considering that the majority of patients with post-stroke aphasia were characterized by unilateral infarction lesions, we inputted individual lesion maps and set the *Enantiomorphic Normalization* option to replace the lesion with the tissue from the intact hemisphere in order to improve the normalized results. This is an alternative non-linear registration method that utilizes the between-hemispheric enantiomorphic relation to correct the signal within the lesion, using information from the undamaged homologous region within the contralesional hemisphere (Nachev et al., 2008). It has advantages over traditional cost function masking, by reducing the steeply increased normalization error as the lesion size increases. For the controls, the *New Segment* module in SPM12 software (Penny et al., 2007) and *DARTEL* (Ashburner, 2007) were used. Visual inspection was performed to check the quality of normalization in both groups.

#### Arterial Spin Labeling Preprocessing

Arterial spin labeling difference images were generated by subtracting the label images from the control images. After averaging three ASL difference images, CBF images were obtained using proton density-weighted reference images (Xu et al., 2010). With the deformation information derived from T1-weighted structural image preprocessing, individual ASL images were transformed into the standard space using SPM12 software. Subsequently, all co-registered individual CBF maps were standardized by dividing the mean CBF value within the gray mask. The standardized CBF maps were resized to 3 mm × 3 mm × 3 mm voxels and then smoothed with a Gaussian kernel of 6-mm full width at half maximum (FWHM).

#### fMRI Preprocessing

DPABI toolbox (Yan et al., 2016) in MATLAB R2013b was utilized for fMRI images. More specifically, the key steps were as follows: (1) removal of the first 10 volumes of individual images to reach magnetization equilibrium and saturation effects; (2) time correction for the acquisition time delay among different slices; (3) head motion correction *via* realignment and excluding participants of excess head motion; (4) calculation of the frame-wise displacement to measure volume-to-volume changes in head position (Power et al., 2012); (5) nuisance covariates regression; (6) Friston-24 parameter regression; (7) removal of linear detrend and temporal bandpass filter (0.01–0.08 Hz) (Foerster et al., 2005); (8) spatial normalization with transformation matrices from segmentation of high-resolution structural images; (9) reslicing normalized fMRI images into 3-mm cubic voxels; and (10) Gaussian smoothing (FWHM = 6 mm).

#### Voxel-Mirrored Homotopic Connectivity Calculations

First, a study-specific symmetrical template was generated by averaging normalized T1-weighted images and the mirrored counterpart. Second, a symmetrical template was used to refine the non-linear registration to the MNI space for each participant. Subsequently, a refined transformation was applied to the symmetrical brain template. Homotopic connectivity was calculated as Pearson’s correlation of time series between each pair of the symmetric interhemispheric voxel. The threshold of Pearson’s correlation coefficient was set at *r* = 0.2 in order to minimize weak correlations potentially caused by the background noise (Zhu et al., 2017). Afterward, Fisher *r*-to-*z* transformation was performed to increase distribution normality. Finally, VMHC maps were acquired.

#### Cerebral Blood Flow-Voxel-Mirrored Homotopic Connectivity Coupling Analysis

Correlation analyses across voxels within the mask of the whole gray matter were performed to quantify the neurovascular characteristics of corresponding voxels at the global gray matter level using the *spatial correlation* function of Image Calculator in DPABI (Liang et al., 2013). To quantify the level of blood supply per unit of homotopic connectivity strength, the CBF/VMHC ratio was computed by dividing the original values of voxel-wise CBF with VMHC using Image Calculator in DPABI (Liang et al., 2013). All CBF, VMHC, and CBF/VMHC ratio maps were normalized by subtracting the average value and dividing by the standard deviation (SD) value of voxels in the mask of the gray matter (Zhu et al., 2017). Thus, *z*-score maps of CBF, VMHC, and CBF/VMHC ratio were generated.

### Statistical Analysis

Between-group whole-brain differences in voxel-wise VMHC, CBF, and CBF/VMHC ratios were compared using a two-sample *t*-test in SPM12 software. The binary inverted lesion masks for individual images were used to ameliorate the influence of the damaged area on the voxel-wise statistics. Additionally, to provide voxel-wise comparisons of stable performance, we excluded voxels from statistical analyses that have too many lesions at the group level (>50% of the sample) (Supplementary Figure 1). A voxel-wise false discovery rate (FDR) method was utilized with a corrected *p* < 0.05. For region of interest-based analyses, general linear models were used to compare between-group differences in the mean values of regions with significant clusters using SPSS Statistics (version 26). Pearson’s partial correlation analysis was conducted to examine the associations between auditory comprehension scores and imaging metrics in the regions of significant differences defined by the Brainnetome Atlas (Fan et al., 2016). The threshold for multiple comparisons (eight subregions for the VMHC, two for the CBF, and six for the CBF/VMHC ratio) was FDR-corrected (corrected *p* < 0.05) (Benjamini and Hochberg, 1995; Genovese et al., 2002). Nuisance variables (age, education level, sex, and lesion size) were controlled for between-group comparisons and imaging-behavioral correlation analyses.

## Results

### Demographics and Clinical Features

**FIGURE 1:**
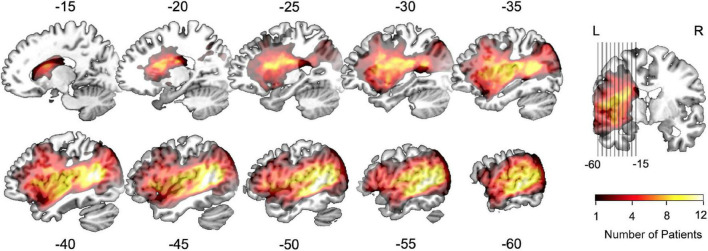
Lesion overlap map of the aphasic group (*n* = 19). The colored voxels depict left-hemisphere lesion distributions on the standard MNI-152 template. The color bar denotes the number of patients with a lesion in a given voxel between 1 and 12.

Clinical profiles of participants are provided in Table 1. The post-stroke time in the included aphasic patients was 11.5 ± 5.7 days, and the mean lesion volume was 61.0 ± 56.7 mL. The average age was 60.1 ± 13.1 years for patients and 54.3 ± 13.7 years for healthy participants. The education level was 9.9 ± 3.7 years for patients and 10.4 ± 4.5 years for healthy participants. The proportion of females was 26.3% among patients and 45.5% among healthy participants. No significant between-group differences in these demographic factors were detected (all *p* > 0.05). Moreover, there was no significant difference in intracranial volume between patients and controls in our cohort (*p* > 0.05). The average AQ measuring the overall severity of language deficits was 38.8 ± 23.2 for patients with aphasia. The total speech comprehension score was 55.4 ± 23.8%, and the average performance of the subdomains was 54.2 ± 27.3% for word-level speech comprehension and 55.1 ± 21.7% for sentence-level speech comprehension. The group-specific distribution of ischemic lesions is shown in the lesion overlap map in Figure 1.

### Spatial Distributions of Neurovascular Changes

**FIGURE 2:**
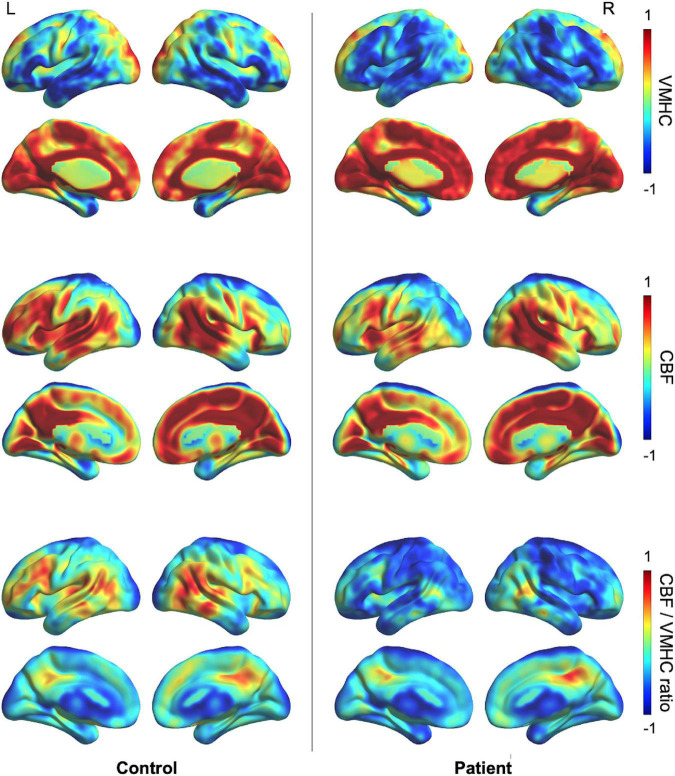
Group-specific averaged maps showing the spatial distribution of VMHC, CBF, and the CBF/VMHC ratio. The VMHC, CBF, and CBF/VMHC ratio maps were normalized to *z*-scores and averaged across participants within groups. CBF, cerebral blood flow; VMHC, voxel-mirrored homotopic connectivity.

Spatial distributions of VMHC, CBF, and CBF/VMHC ratios are shown in Figure 2. A decrease in VMHC was observed in the bilateral perisylvian regions and left posterior occipital cortex, and the lower CBF was mostly located in the left perisylvian and prefrontal regions in patients with post-stroke aphasia. The CBF per unit homotopic connectivity strength (CBF/VMHC ratio) in the control group was greater in the left peri-Wernicke temporoparietal areas and middle frontal gyrus.

### Voxel-Wise Neurovascular Comparisons

**FIGURE 3:**
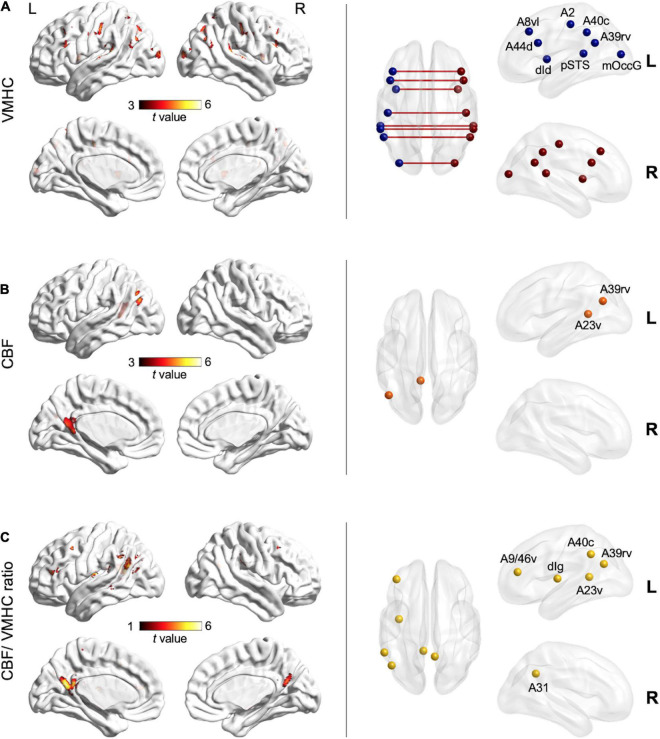
Voxel-wise neurovascular comparisons between the aphasic group and control group (FDR-corrected *p* < 0.05) after controlling for age, sex, education level, and lesion size. Left column: clusters of warm colors indicate significant decreases in VMHC **(A)**, CBF **(B)**, and the CBF/VMHC ratio **(C)** in aphasia, respectively, with lighter colors denoting higher *t*-values and significance. Right column: nodes indicate subregions of the Brainnetome Atlas corresponding to significant clusters. A2, area 2; A23v, ventral area 23; A31, area 31; A39rv, rostroventral area; A40c, caudal area 40; A44d, dorsal area 44; A8vl, ventrolateral area 8; A9/46v, ventral area 9/46; CBF, cerebral blood flow; dId, dorsal dysgranular insula; dIg, dorsal granular insula; mOccG, middle occipital gyrus; pSTS, posterior superior temporal sulcus; VMHC, voxel-mirrored homotopic connectivity.

Further whole-brain voxel-level comparisons revealed significant clusters (FDR-corrected *p* < 0.05) (Figure 3 and Supplementary Table 1). In post-stroke aphasia, interhemispheric homotopic connectivity was significantly reduced in dorsal area 44 of the inferior frontal gyrus, ventrolateral area 8 of the middle frontal gyrus, pSTS, dorsal dysgranular insula, area 2 of the postcentral gyrus, rostroventral area 39 (A39rv), caudal area 40 (A40c) of the inferior parietal lobule, and middle occipital gyrus (Figure 3A). As compared with controls, left ventral area 23 of the cingulate gyrus (A23v) and A39rv showed significantly decreased regional CBF (Figure 3B), whereas lower CBF/VMHC ratios were observed in left ventral area 9/46 of the middle frontal gyrus, dorsal granular insula, A23v, A39rv, A40c, and right area 31 of the precuneus in aphasic patients (Figure 3C).

### Cerebral Blood Flow, Voxel-Mirrored Homotopic Connectivity, and Neurovascular Correlations for the Whole Gray Matter

Group comparisons of the mean metrics for the whole gray matter are shown in Supplementary Figure 2. The VMHC of the whole gray matter indicated that interhemispheric homotopic connectivity was reduced in the patient group (0.39 ± 0.10 in patients vs. 0.53 ± 0.04 in controls, *t* = 5.347, *p* < 0.001). Non-significant reduction in the mean CBF of the whole gray matter was observed between two groups (43.8 ± 14.8 mL/100 g/min in patients vs. 45.8 ± 10.0 mL/100 g/min in controls, *t* = 0.492, *p* > 0.05). Additionally, the CBF-VMHC correlation in the whole gray matter was reduced in aphasic patients (0.44 ± 0.06 in patients vs. 0.51 ± 0.09 in controls, *t* = 3.227, *p* = 0.003).

### Relationships Between Neurovascular Metrics and Speech Comprehension

**FIGURE 4:**
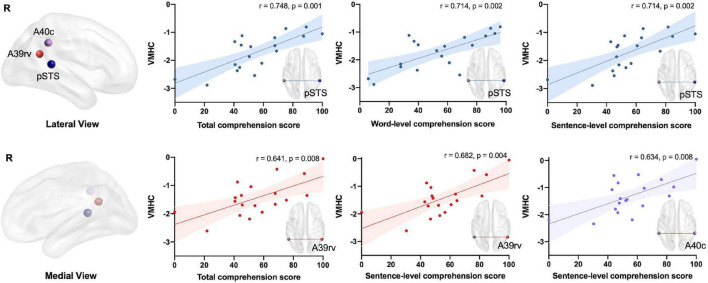
Associations between speech comprehension and regional interhemispheric homotopic connectivity in patients with post-stroke aphasia after controlling for age, sex, education level, and lesion size (FDR-corrected *p* < 0.05). Colors indicate different brain regions: blue for pSTS, red for A39rv, and purple for A40c. A39rv, rostroventral area; A40c, caudal area 40; pSTS, posterior superior temporal sulcus; VMHC, voxel-mirrored homotopic connectivity.

**FIGURE 5:**
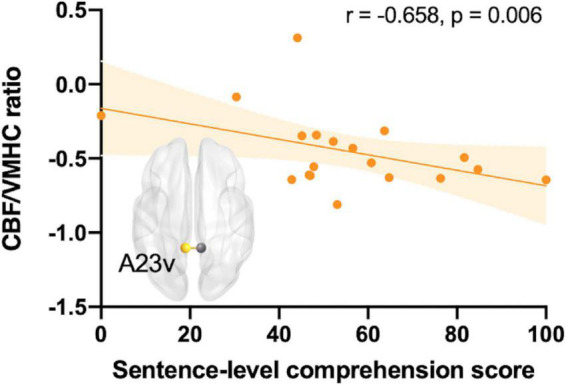
Correlation between sentence-level speech comprehension subdomains and the regional mean CBF/VMHC ratio for the cingulate gyrus subregion (A23v) in patients with post-stroke aphasia after controlling for age, sex, education level, and lesion size (FDR-corrected *p* < 0.05). A23v, ventral area 23; CBF, cerebral blood flow; VMHC, voxel-mirrored homotopic connectivity.

Regional interhemispheric homotopic connectivity significantly correlated with speech comprehension ability in the peri-Wernicke area (Figure 4). Significantly positive correlations between the overall speech comprehension score and VMHC were observed in the pSTS (*r* = 0.748, *p* = 0.001) and A39rv (*r* = 0.641, *p* = 0.008), respectively (FDR-corrected *p* < 0.05). Regarding the subdomains, the VMHC of the pSTS significantly associated with the single word (*r* = 0.714, *p* = 0.002) or sentence (*r* = 0.714, *p* = 0.002) speech comprehension score (FDR-corrected *p* < 0.05). The VMHC of A39rv and A40c also exhibited significant associations with sentence-level comprehension performance (A39rv: *r* = 0.682, *p* = 0.004; A40c: *r* = 0.634, *p* = 0.008; FDR-corrected *p* < 0.05). Regional CBF did not show any significant or trend-level correlation with speech comprehension (FDR-corrected *p* > 0.05). With respect to neurovascular interactions, only the mean CBF/VMHC ratio in the cingulate gyrus subregion (A23v) exhibited a negative association with sentence-level comprehension, as shown in Figure 5 (*r* = −0.658, *p* = 0.006; FDR-corrected *p* < 0.05).

## Discussion

The present study revealed that both the CBF-VMHC correlation and CBF/VMHC ratio decreased in patients with post-stroke aphasia, particularly in the brain regions including the peri-Wernicke temporoparietal areas. We also identified a significant association between comprehension and VMHC in the peri-Wernicke area, as well as a negative correlation between sentence-level comprehension and CBF/VMHC ratio in the cingulate gyrus subregion. To our knowledge, this study is the first to illustrate CBF-VMHC coupling changes in aphasia. These findings provide some insights into potential neurophysiological mechanisms underlying stroke-induced speech comprehension deficits.

### Stroke-Induced Abnormal Homotopic Connectivity and Neurovascular Alterations

Our study showed that the VMHC in the bilateral perisylvian regions was lower than that in other cortical regions among healthy controls. This finding is supported by previous studies reporting weaker homotopic RSFC in language-related regions (Stark et al., 2008; Zuo et al., 2010), which is consistent with the functional asymmetry known as language lateralization (Price, 2010). Moreover, the VMHC in the bilateral perisylvian regions decreased in post-stroke patients, as compared with that in controls. Notably, reduced interhemispheric RSFC could indicate impaired interhemispheric connection for integrating information (Kelly et al., 2011), especially the declined homotopic communication for language processing after a left-hemisphere stroke in this study.

Perfusion of the left inferior parietal lobule subregion A39rv and cingulate gyrus subregion A23v was reduced. Moreover, left A39rv and A23v also showed decreased blood supply per unit of homotopic connection, demonstrating the neurovascular disturbance underlying the disrupted homotopic connection. A39rv, located within the vicinity of the Wernicke area, tends to be one of the critical subregions subserving semantic processing, and its lesion could lead to transcortical sensory aphasia and impairment in sentence-level comprehension (Kertesz et al., 1982; Dronkers et al., 2004). Although the cingulate gyrus subregion, the other area with a significant difference in the CBF/VMHC ratio, is not a classic language-related region, emerging evidence suggests that it supplements language processing through its contribution to cognitive control (Brownsett et al., 2014; Geranmayeh et al., 2017).

These findings of neurovascular alterations may aid us in understanding the pathophysiology of post-stroke aphasia during the subacute period. The neurovascular unit, which consists of neurons, astrocytes, endothelial cells, and smooth muscle cells or pericytes as the vasomotor apparatus (Iadecola, 2017), serves as the microstructural basis for the linkage between neural activity and brain hemodynamics. Stroke disrupts the highly orchestrated pattern of the neurovascular unit by undermining the signaling pathways and effector systems across the cerebrovascular network; in turn, the neurovascular unit plays crucial roles in ischemic stroke *via* multiple aspects including inflammatory immune response, blood–brain barrier regulation, and neurovascular repair (Wang et al., 2021). Given the difficulty in investigating the association between human language function and neurovascular alterations from the perspective of molecular biology, our study provides invasive surrogate markers from multimodal imaging by combining fMRI and ASL. Neurovascular dissociation might be one of the potential pathophysiological mechanisms underlying subacute post-stroke aphasia.

### Temporoparietal Homotopic Connectivity and Comprehension

It is interesting to note that all regions with significant VMHC-comprehension correlations were located in the temporoparietal region, within the vicinity of the Wernicke area. Anatomically, the generalized Wernicke area includes not only the left posterior STG and supramarginal gyrus (SMG) but also the angular gyrus (AG) (Bogen and Bogen, 1976). The pSTS area is near the posterior STG, whereas A39rv is within the AG and A40c is the SMG subregion. The left pSTS and the SMG subregion A40c are involved in the phoneme perception process, the first stage of speech comprehension (Binder et al., 2000; Hickok and Poeppel, 2007), whereas the AG subregion A39rv is a critical area within the semantic network supporting concept retrieval and conceptual integration (Binder et al., 2009).

Previous studies reported a negative AQ-VMHC correlation in the STG (Yang et al., 2017) and associated aphasia severity with disturbed interhemispheric connectivity within the temporoparietal regions (Siegel et al., 2016). In addition, brain network analysis revealed stroke-induced disrupt of interhemispheric integration, and communication across a set of regions was related to deficits across behavioral domains, including language deficits (Siegel et al., 2016). Moreover, evidence from tractography also showed that temporal interhemispheric connectivity measured using volumes of white matter was a predominant predictor of the overall severity of impaired language performance (Northam et al., 2012). However, few researchers have investigated the association of speech comprehension with homotopic connectivity between peri-Wernicke temporoparietal areas and their homologs.

Our findings indicate a close association between the interhemispheric connection of the Wernicke area and speech comprehension. High-level auditory areas in both hemispheres (i.e., the bilateral STG and adjacent pSTS) are involved in the phonologic retrieval stage; therefore, the connection with the homolog supplementing phonologic retrieval would be abnormal when there are unilateral lesions (Poeppel, 2001). In addition to low-level acoustic and phonetic analyses, functional imaging data analyses suggest that bihemispheric temporal activation is involved in lexical processes (Jung-Beeman, 2005; Bozic et al., 2010). The contribution of strong functional connections linking bilateral superior temporal regions to normal narrative speech comprehension has been revealed by positron emission tomography findings (Warren et al., 2009). Nevertheless, how the bihemispheric temporoparietal regions interact with each other after stroke is yet to be systematically elucidated. A recent fMRI study demonstrated that both excitatory and inhibitory homotopic connections induced by left-hemisphere damage might be maladaptive in post-stroke aphasia because they disrupt the normal interhemispheric coordination and communication (Chu et al., 2018). Structurally, the posterior third of the corpus callosum contains fibers linking homotopic temporoparietal regions and has been considered to be the crucial interhemispheric structure for the interaction of prosodic and syntactic information during the speech comprehension process (Friederici et al., 2007).

### Contribution of Cingulate Neurovascular Characteristics to Comprehension

The contribution of neurovascular characteristics to brain connectivity changes revealed in this study raises intriguing questions about the role of domain-general subregions, including the cingulate cortex, in speech comprehension. Our results indicate that the neurovascular multimodal index can determine potential functionally implicated regions underlying the network architecture of speech comprehension. Notably, while the VMHC exhibited a correlation with comprehension in regions surrounding the Wernicke area, the neurovascular metric CBF/VMHC in the cingulate cortex, but not the functional metric VMHC, was related to comprehension. Recently, the clinical utility of the Wernicke area has been challenged because the distributed networks extending beyond the so-called Wernicke area are involved in semantic processing (Tremblay and Dick, 2016). Considering that speech comprehension is a distributed language domain rather than a single process, the region supporting comprehension is a large-scale network rather than a localized center (Binder, 2017). A comprehensive meta-analysis showed that the network implicated in semantic processing could be classified into three categories, and one of the subsystems was the medial paralimbic area, including the posterior cingulate gyrus (Binder et al., 2009). This finding supports the contribution of the posterior cingulate gyrus to linguistic processing, subserving the semantic retrieval as an interface related to episodic encoding systems (Binder et al., 2009), with its close reciprocal connections with the hippocampus *via* the cingulum bundle (Kobayashi and Amaral, 2007). Despite the left-lateralized feature of the semantic system, the activation pattern of the posterior cingulate gyrus and AG is bilateral (Binder et al., 2009), suggesting the involvement of the bihemispheric homologs. Moreover, the cingulate cortex may contribute to language through its broad role in learning and cognition within the cingulo-opercular network, facilitating post-stroke language recovery (Brownsett et al., 2014; Geranmayeh et al., 2014, 2017).

### Decompositional Substrate for Speech Comprehension Subdomains

Despite the growing consensus on the phonological and lexical aspects of speech comprehension, there is still no general agreement regarding the critical regions at the sentence level. Our study sheds light on the correlations between word- and sentence-level speech comprehension and functional brain activity. While the VMHC of the pSTS correlated with all speech comprehension subdomains, the VMHC of the AG subregion A39rv and the SMG subregion A40c, along with the CBF/VMHC ratio in the left cingulate gyrus subregion A23v, showed specific associations with sentence-level comprehension. Notably, we identified that the shared attribute of all sentence-specific regions plays a role in high-level integrative processes. This is consistent with the requirement for multiple-level processing involved in auditory sentence-level comprehension, including the coordination among phonological perception, lexical-semantic processing, syntax, and high-level cognitive functions (e.g., attention, verbal working memory, and cognitive control) (Adezati et al., 2022). Aside from the posterior cingulate gyrus integrating episodic memory with semantic retrieval, the AG at the temporoparietal junction subserves the top processing hierarchy requiring conceptual combination, including sentence-level comprehension (Xu et al., 2005). In contrast, the pSTS is the neural substrate for the earlier stage of comprehension (i.e., phoneme perception for spoken words), the damage to which tends to correlate with impairment in all comprehension subdomains (Liebenthal et al., 2005; DeWitt and Rauschecker, 2012). Nevertheless, considering the lack of high-level cognitive measures in the current study, these decompositional characteristics for speech comprehension subdomains should be interpreted with caution.

### Limitations

The study cohort has a relatively small sample size. Additional insights may be obtained in future studies with more statistical power. The small number of fMRI volumes limited the quality of imaging and interpretability of findings. Considering that CBF and VMHC are indirect metrics of perfusion status and homotopic neural activity, respectively, their neurobiological implications should be interpreted with caution. Moreover, VMHC merely computes the structural homotopic areas. A novel method investigating connectivity between functionally homotopic voxels may provide better interpretability of inter-hemisphere connectivity (Sun et al., 2022) and could be utilized in further studies. Additionally, the language battery was not detailed enough to explore the comprehension domain in depth, and the scales for other cognitive domains were lacking in this study. For instance, given that the sentence-level processing task requires more attention support than the single-word task, the conclusion for the comprehension subdomains would have better interpretability by regressing the influence from the severity of cognitive control impairment. Specific regions for the comprehension subdomains necessitate further discrimination in future studies.

## Conclusion

This study suggests that the perfusion level per unit homotopic connectivity strength was reduced in brain regions, including the left peri-Wernicke temporoparietal areas and the cingulate gyrus subregion, in patients with post-stroke aphasia. Speech comprehension deficits were associated with homotopic connectivity in the vicinity of the temporoparietal areas. Our findings suggest that abnormal neurovascular characteristics in the cingulate gyrus subregion, which might be associated with impairment in semantic integration, could be one of the key mechanisms underlying the sentence-level comprehension deficits in stroke-induced aphasia.

## Data Availability Statement

The raw data supporting the conclusions of this article will be made available by the authors, without undue reservation.

## Ethics Statement

The studies involving human participants were reviewed and approved by the Institutional Ethics Committee of The First Affiliated Hospital of Zhejiang University School of Medicine. The patients/participants provided their written informed consent to participate in this study.

## Author Contributions

JZ was involved in the study design, data processing, and manuscript drafting. JY and DS contributed to data acquisition and preprocessing. ScZ participated in data processing and statistical analysis. YL was responsible for drafting the manuscript and figures. YY contributed to data acquisition and analysis. LZ participated in statistical analysis, drafting, and revision of figures. WZ contributed to data interpretation, manuscript revision, and funding acquisition. SsZ participated in statistical analysis and data interpretation. FH contributed to data acquisition and study supervision. XY contributed to study supervision, manuscript revision, and funding acquisition. BL was responsible for the study concept and manuscript revision. All authors contributed to the article and approved the submitted version.

## Conflict of Interest

The authors declare that the research was conducted in the absence of any commercial or financial relationships that could be construed as a potential conflict of interest.

## Publisher’s Note

All claims expressed in this article are solely those of the authors and do not necessarily represent those of their affiliated organizations, or those of the publisher, the editors and the reviewers. Any product that may be evaluated in this article, or claim that may be made by its manufacturer, is not guaranteed or endorsed by the publisher.

## Footnotes

1
http://www.itksnap.org/

2
https://www.nitrc.org/projects/clinicaltbx/
