# Supplementary material for: Altered Coupling Between Cerebral Blood Flow and Voxel-Mirrored Homotopic Connectivity Affects Stroke-Induced Speech Comprehension Deficits
Source: Front Aging Neurosci. 2022 Jun 23;14:922154. doi: 10.3389/fnagi.2022.922154 (PMC9260239; doi:10.3389/fnagi.2022.922154)
Supplement: Supplementary file 1 [file Data_Sheet_1.PDF]

Altered Coupling Between Cerebral Blood Flow and Voxel-Mirrored Homotopic Connectivity Affects Stroke-Induced Speech Comprehension Deficits

Jie Zhang[1,2], Desheng Shang[3], Jing Ye[1], Yi Ling[2], Shuchang Zhong[1], Shuangshuang Zhang[1], Li Zhang[1], Yamei Yu[4], Fangping He[2], Xiangming Ye[1*], Benyan Luo[2,5*]

[1]Center for Rehabilitation Medicine, Rehabilitation Research Institute of Zhejiang Province, Department of Rehabilitation Medicine, Zhejiang Provincial People's Hospital (Affiliated People's Hospital, Hangzhou Medical College), Hangzhou, China

[2]Department of Neurology & Brain Medical Center, The First Affiliated Hospital of Zhejiang University School of Medicine, Hangzhou, China

[3]Department of Radiology, The First Affiliated Hospital of Zhejiang University School of Medicine, Hangzhou, China

[4]Department of Neurology, Sir Run Run Shaw Hospital, Zhejiang University School of Medicine, Hangzhou, China

[5]Collaborative Innovation Center for Brain Science, Zhejiang University School of Medicine, China

*** Correspondence:**
Xiangming Ye
yexmdr@hotmail.com

Benyan Luo
luobenyan@zju.edu.cn

Supplementary Material

1 Supplementary Figures

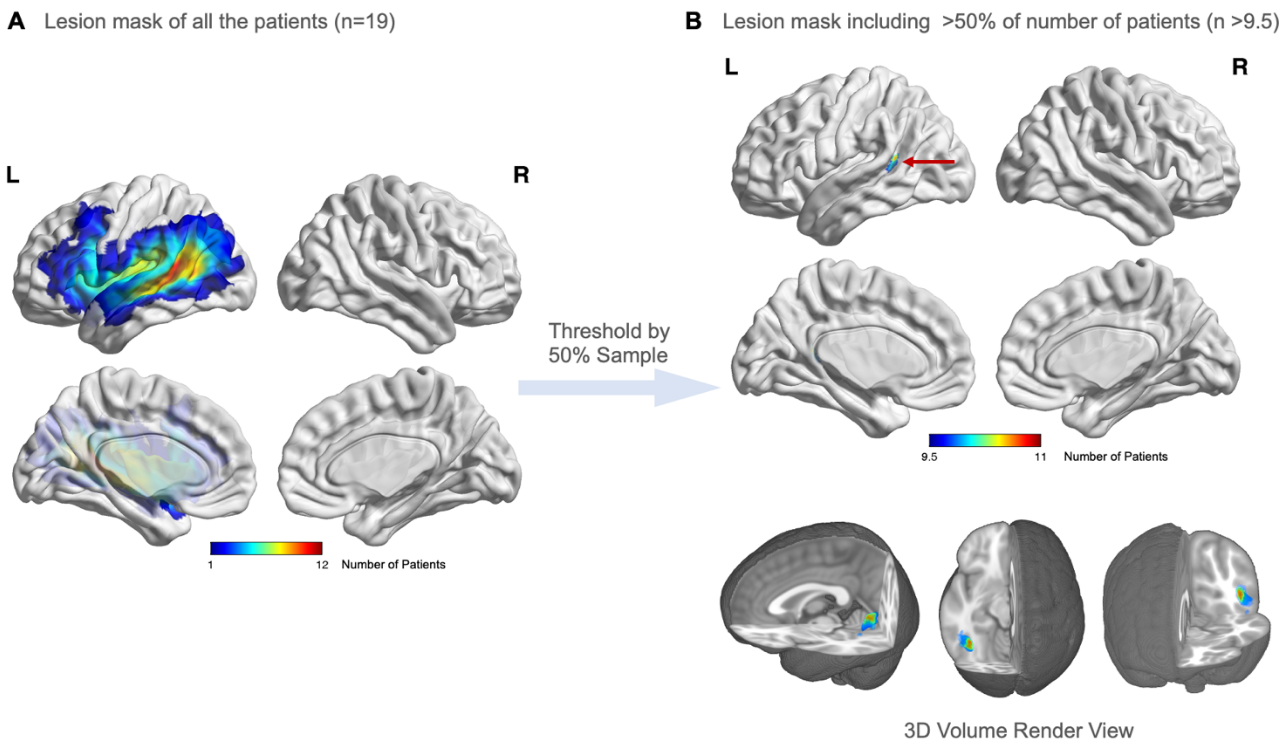

Supplementary Figure 1. Lesion masks of different thresholds of number of patients. (A) Lesion mask of all the patients (n=19); (B) Lesion mask including lesioned voxels >50% of number of patients (n >9.5), which is excluded from group-level voxel-wise statistical analyses.

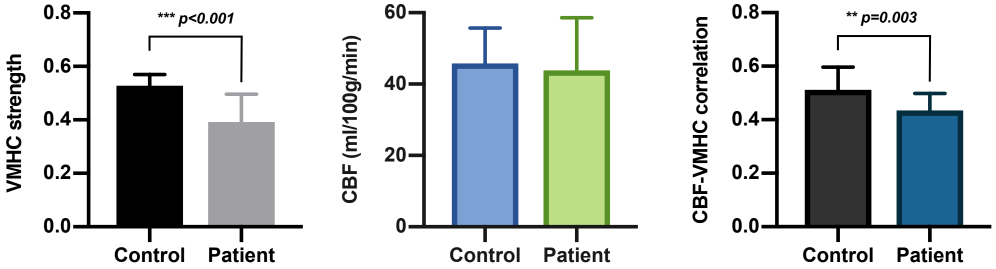

Supplementary Figure 2. Whole gray matter level VMHC, CBF, and CBF-VMHC coupling alterations in post-stroke aphasia. Both the mean VMHC and CBF-VMHC correlation coupling of whole gray matter are significantly reduced in patients compared with healthy participants. The mean CBF between patients and controls did not show significant differences. CBF: cerebral blood flow; VMHC: voxel-mirrored homotopic connectivity. ***: $p < 0.001$; **: $0.001 < p < 0.01$.

2 Supplementary Tables

Supplementary Table 1. Whole-brain neurovascular differences between the aphasic group and control group (FDR-corrected $p < 0.05$) after controlling for age, sex, education level, and lesion size.

**Metrics**	**Hemisphere**	**BNA-based Regions**	**Cluster size (Voxel number)**	**MNI coordinates**			**Peak *t* value**
				x	y	z	
VMHC	Bilateral	A39rv	235	-48	-66	30	5.4249
	Bilateral	pSTS	206	-52	-50	11	5.8261
	Bilateral	mOccG	154	-31	-89	11	5.5645
	Bilateral	A40c, A2	124	-46	-30	50	5.1243
	Bilateral	A8vl, A44d	87	-42	24	40	5.1115
	Bilateral	dId	86	-42	6	6	5.2077
CBF	Left	A39rv	561	-38	-70	34	5.9838
	Left	A23v	416	-6	-46	6	4.9069
CBF/VMHC ratio	Left	A23v	156	-8	-47	10	6.3457
	Right	A31	69	2	-58	22	5.1589
	Left	A39rv	65	-38	-70	32	7.0069
	Left	dIg	25	-38	-16	14	5.3379
	Left	A40c	25	-58	-48	34	5.39
	Left	A9/46v	17	-44	42	16	5.1825

Notes: A2, area 2; A23v, ventral area 23; A31, area 31; A39rv, rostroventral area; A40c, caudal area 40; A44d, dorsal area 44; A8vl, ventrolateral area 8; A9/46v, ventral area 9/46; CBF, cerebral blood flow; dId, dorsal dysgranular insula; dIg, dorsal granular insula; mOccG, middle occipital gyrus; pSTS, posterior superior temporal sulcus; VMHC, voxel-mirrored homotopic connectivity.
